# Detoxification of Toxic Phorbol Esters from Malaysian *Jatropha curcas* Linn. Kernel by *Trichoderma* spp. and Endophytic Fungi

**DOI:** 10.3390/ijms15022274

**Published:** 2014-02-05

**Authors:** Azhar Najjar, Norhani Abdullah, Wan Zuhainis Saad, Syahida Ahmad, Ehsan Oskoueian, Faridah Abas, Youssuf Gherbawy

**Affiliations:** 1 Department of Microbiology, Faculty of Biotechnology and Biomolecular Sciences, Universiti Putra Malaysia, Serdang 43400, Selangor, Malaysia; E-Mails: az-najjar@hotmail.com (A.N.); zuhainis@upm.edu.my (W.Z.S.); ehs424@yahoo.com (E.O.); 2 Department of Biochemistry, Faculty of Biotechnology and Biomolecular Sciences, Universiti Putra Malaysia, Serdang 43400, Selangor, Malaysia; E-Mail: syahida@upm.edu.my; 3 Institute of Tropical Agriculture, Universiti Putra Malaysia, Serdang 43400, Selangor, Malaysia; 4 Department of Food Science, Faculty of Food Science and Technology, Universiti Putra Malaysia, Serdang 43400, Selangor, Malaysia; E-Mail: faridah_abas@upm.edu.my; 5 Department of Biology, Faculty of Science, Taif University, P.O. Box: 888-Taif, Taif 21974, Saudi Arabia; 6 Botany Department, Faculty of Science, South Valley University, Qena 83523, Egypt; E-Mail: youssufgherbawy@yahoo.com

**Keywords:** phorbol esters detoxification, phorbol esters-rich fraction utilization, fungal treatment, mycelial dry weight, cytotoxicity, cell lines

## Abstract

The presence of phorbol esters (PEs) with toxic properties limits the use of *Jatropha curcas* kernel in the animal feed industry. Therefore, suitable methods to detoxify PEs have to be developed to render the material safe as a feed ingredient. In the present study, the biological treatment of the extracted PEs-rich fraction with non-pathogenic fungi (*Trichoderma harzianum* JQ350879.1, *T. harzianum* JQ517493.1, *Paecilomyces sinensis* JQ350881.1, *Cladosporium cladosporioides* JQ517491.1, *Fusarium chlamydosporum* JQ350882.1, *F. chlamydosporum* JQ517492.1 and *F. chlamydosporum* JQ350880.1) was conducted by fermentation in broth cultures. The PEs were detected by liquid chromatography-diode array detector-electrospray ionization mass spectrometry (LC-DAD-ESIMS) and quantitatively monitored by HPLC using phorbol-12-myristate 13-acetate as the standard. At day 30 of incubation, two *T. harzianum* spp., *P. sinensis* and *C. cladosporioides* significantly (*p* < 0.05) removed PEs with percentage losses of 96.9%–99.7%, while *F. chlamydosporum* strains showed percentage losses of 88.9%–92.2%. All fungal strains could utilize the PEs-rich fraction for growth. In the cytotoxicity assay, cell viabilities of Chang liver and NIH 3T3 fibroblast cell lines were less than 1% with the untreated PEs-rich fraction, but 84.3%–96.5% with the fungal treated PEs-rich fraction. There was no inhibition on cell viability for normal fungal growth supernatants. To conclude, *Trichoderma* spp., *Paecilomyces* sp. and *Cladosporium* sp. are potential microbes for the detoxification of PEs.

## 1. Introduction

*Jatropha curcas* Linn. belongs to the family *Euphorbiaceae*, which was originally native to South America but is now found in abundance in South and Central America, Africa and Asia. Different parts of the plant have been shown to possess biological activities that could be associated with the presence of phenolics, terpenoids and flavonoids [1]. The main interest in this plant is as a source of kernel oil. Ripe kernel contains about 60% oil, which can be converted to biodiesel. However, previous studies have shown that *J. curcas* kernel contained considerable levels of phorbol esters (PEs). These esters were found to have carcinogenic and mutagenic properties [2]. The amount of PEs in the kernel oil varies. The Malaysian *J. curcas* seed oil had a lower content (0.23%) than that of seed oil from India and Indonesia which had 0.58% and 1.58%, respectively [3].

Several approaches to eliminate PEs from both *Jatropha* kernel and oil have been reported. Physiochemical treatments of *Jatropha* meal could only reduce the level of PEs to 76% [4]. Organic solvents however have been used to detoxify PEs in defatted *Jatropha* kernel meal up to an undetectable level [5]. Unfortunately these procedures are not only costly, but also have some negative impacts on the environment. The physiochemical procedure is a non-specific process which is based on combination of heat and chemical applications to eliminate PEs from *Jatropha* kernel [6].

Removal of PEs by microbial treatment is largely dependent on the types of microbes, incubation periods and substrates used [7,8]. The complete removal of PEs from *J. curcas* seed cake was observed after nine days incubation with *Pseudomonas aeruginosa* [1]. Also, a decrease of 20% of the concentration of PEs in seed cake of *J. curcas* was observed in the treatment with *Ganoderma resinaceum*, but up to 91% and 97% in the treatments with *Bjerkandera adusta* and *Phlebia rufa*, respectively, after 30 days [6]. Although, these studies showed potential removal of PEs following microbial treatments, the pathogenicity of some microorganisms limit their applications in the detoxification of *Jatropha* kernel. Thus, safer microorganisms have to be considered in treating *Jatropha* kernel in the production of an edible feed source. In recent years, there has been an increasing interest in some non-pathogenic and non-toxic *Ascomycota* fungi, particularly *Trichoderma* genera and endophytic fungi, such as *Cladosporium*, *Fusarium* and *Paecilomyces* for the degradation of complex organic compounds in soil. For instance, *T. harzianum* has been used to degrade the cellulose and lignin from olive pomace within 50 days [9]. *Cladosporium cladosporioides* has been shown to degrade and transform aquatic humic substances by producing laccase enzyme [10].

Many species of *Trichoderma* and endophytes are widely used for numerous agricultural, medical, and pharmaceutical applications [11,12]. However, these fungi have not been evaluated for detoxifying the PEs present in *Jatropha* kernel. Hence, the present study was focused on *T. harzianum*, *P. sinensis*, *C. cladosporioides* and *F. chlamydosporum* strains in the degradation of PEs from *J. curcas* kernel.

## 2. Results and Discussion

### 2.1. Detection of PEs-Rich Fraction

**Figure 1.**
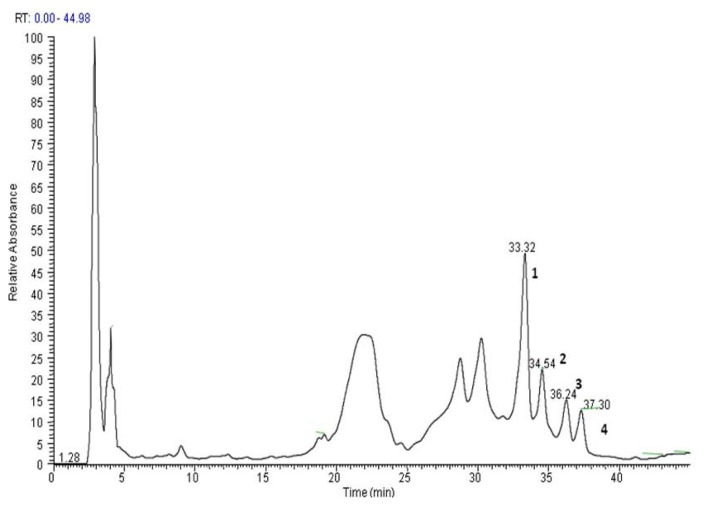
Total ion chromatogram (TIC) by negative ion mode electrospray ionization mass spectrometry (ESI/MS) of the phorbol esters (PEs)-rich fraction. Peaks 1–4 indicate the four derivatives of PE at retention times between 33.32 and 37.30 min.

**Figure 2.**
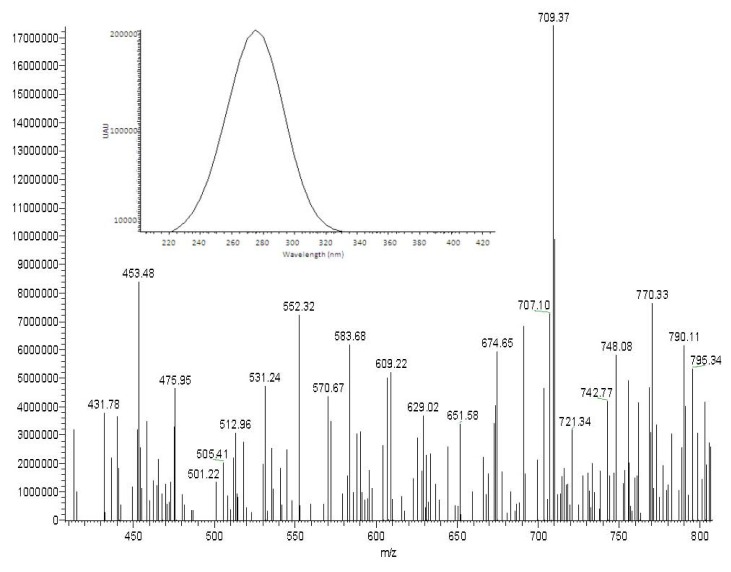
The deprotonated molecular ion [M − H]^−^ spectrum and UV chromatogram (UV λ_max_ at 280 nm) of Peak 1.

The PEs-rich fraction obtained from *Jatropha* kernel was analyzed by liquid chromatography-diode array detector-electrospray ionization mass spectrometry (LC-DAD-ESIMS) to detect the presence of PEs in the fraction. As shown in Figure 1, the total ion chromatogram (TIC) profile showed four peaks at retention times between 33.32 and 37.30 min, corresponding to four derivatives of PE [1]. The deprotonated molecular ion [M − H]^−^ spectrum and UV chromatogram (UV λ_max_ at 280 nm) of Peak 1 is shown in Figure 2. The mass value of Peak 1 was *m*/*z* 709 [M − H]^−^, which corresponds to 12-deoxy-16-hydroxyphorbol, that was reported to be *m*/*z* 711.5 [M + H]^+^ by positive ion mode [1]. Peaks 2–4 are PE compounds with the same diterpene moiety, namely, 12-deoxy-16-hydroxyphorbol [13]. In the present study, only four PE derivatives were detected, whereas in previous studies, six derivatives of PEs in *J. curcus* kernel were reported [13,14]. Similarly, two and five PE derivatives have been observed from different varieties of *J. curcas* [1,3]. It has been suggested that the number of PE derivatives present in *J. curcas* depends on analytical techniques, variety, soil and climatic conditions.

### 2.2. Phorbol Esters Degradation by Fungal Strains

The amount of PEs in the rich fraction obtained from the seed kernel was 66.08 mg PMA equivalent/g dry matter of the PEs-rich fraction. This value is equivalent to 2.78 mg of PEs/g dry matter of kernel. The levels of PEs may vary according to the samples analyzed. A value of 2.79 mg/g kernel has been reported [15], but earlier reports indicated the levels of PEs in different provenances of *J. curcas* to be in the range of 0.8–3.3 mg/g kernel [16].

**Figure 3.**
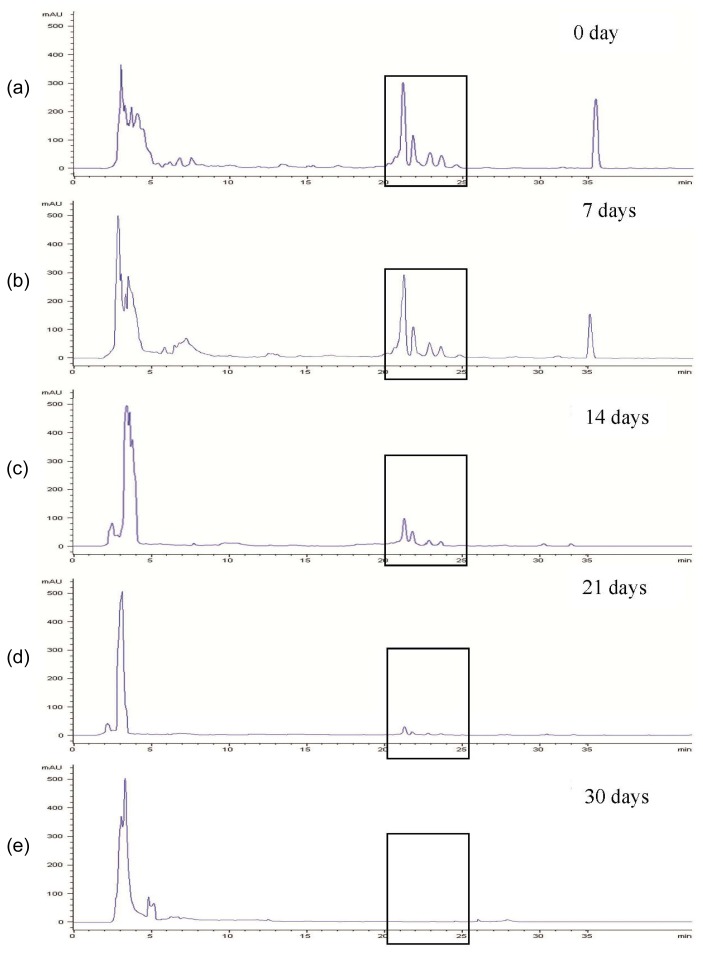
The HPLC chromatogram of phorbol esters treated with *T. harzianum* JQ350879.1 at different incubation periods.

The levels of PEs in the extract of the control flasks (samples without fungal strains) were similar at all incubation times (7, 14, 21 and 30 days). The original levels of PEs were maintained, while extracts from fungal treated flasks showed significant (*p* < 0.05) reduction of PEs. Figure 3a shows a representative of an HPLC elution profile of PEs from *J. curcas* treated with *T. harzianum* JQ350879.1. The four peaks eluted at 21.8, 22.4, 23.4 and 24.3 min under the specified running conditions corresponded to the four different PE derivatives. The retention times of PEs are different according to the techniques used. Analysis by HPLC gave shorter retention times compared to that of LC-DAD-ESIMS analysis. However, the two analytical techniques revealed similar findings, in that only four PE derivatives were detected.

As shown in Figure 3b–e, the levels of PEs decreased gradually with increasing incubation periods. At 30 days of incubation, PEs peaks were no longer visible, indicating almost complete removal of PEs in the culture medium. The percentage of reduction of PEs at the different incubation periods is shown in Table 1. At different incubation periods, the levels of PEs decreased significantly (*p* < 0.05) with *T. harzianum* strains, *P. sinensis* and *C. cladosporioides* showing higher percentage losses than that of *F. chlamydosporum* strains. The levels of PEs at 7 days of incubation in *T. harzianum* strains, *P. sinensis* and *C. cladosporioides* cultures were reduced by 28.4%, 23.5%, 25.0% and 16.5%, respectively, and by 13.7%, 11.5% and 13.2% in the treatments with *F. chlamydosporum* strains, respectively. The same trend in percentage losses was observed at 14 and 21 days, but with higher values. At 30 days of incubation, PE losses were 99.7%, 99.5%, 99.0% and 96.4% for treatment with *T. harzianum* strains, *P. sinensis* and *C. cladosporioides*, respectively. The results clearly demonstrated that *T. harzianum* strains and *P. sinensis* could degrade PEs almost completely. Previous studies have reported the ability of these fungal strains to produce enzymes that could degrade tigliane diterpene esters and other closely related esters [1]. Phorbol esters are compounds with fatty acid moieties esterified to the hydroxyl groups of the tigliane diterpenes. Enzymes such as lipases and esterases are involved in hydrolyzing these ester bonds [6]. Fungal strains like those used in the present study have been reported to produce lipases [9,17,18] and esterases [9,19,20] that in high probability hydrolyze the PEs to fatty acids and the phorbol moiety.

It is essential for industrial and agricultural applications to remove the PEs which are the main toxic compounds present in *Jatropha* kernel for safe and maximum utilization. It has been reported that the PE level in the *Jatropha* kernel of between 0.02 and 0.11 mg/g is considered safe for human and animal consumption [6]. In the present study, the level of PEs has been reduced by 99%, equivalent to a level of 0.02 mg/g of PEs remaining in the kernel. Accordingly, this level of PEs is considered safe as a feed resource.

A number of studies have been conducted to evaluate the effects of treated *Jatropha* cake (defatted kernel) in feeding trial experiments in goats, fish and poultry. Treated *Jatropha* cake with 77% of PE reduction by *Aspergillus niger* and *Penicillium* sp. could replace 50% of soybean meal in the goat diet [21]. It was also observed that fish growth performance increased five times in the group fed with detoxified PEs in *Jatropha* kernel meal by physiochemical treatment [5]. Recently, it has been suggested that *Jatropha* seed cake can be an alternative feed for broiler after PE detoxification of 79.7% through biological treatment with *A. niger* and *Neurospora sitophila* [22]. These reports nevertheless, indicate the possibility of using treated *Jatropha* cake as a feed ingredient. Although, *Jatropha* seed also contained other anti-nutritional compounds like trypsin inhibitor, lectins, phytate and saponins, they could be easily destroyed by heat [4].

### 2.3. Utilization of PEs-Rich Fraction as Carbon Source for Fungal Growth

**Figure 4.**
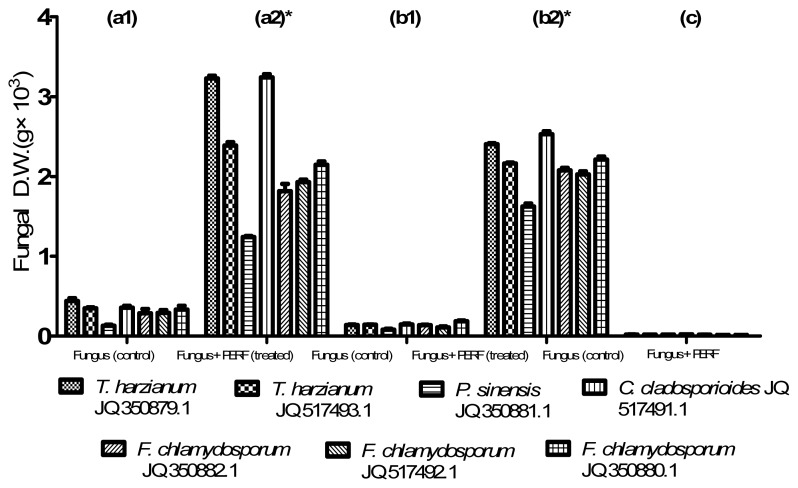
Dry weight (DW) of fungal strains grown in (**a1**) PDB; (**a2**) PDB + phorbol esters-rich fraction (PERF); (**b1**) Mineral salts broth (MSB); (**b2**) MSB + PERF and (**c**) PERF only after 30 days incubation. Treated media contained 2 g of PERF. The standard error bars were calculated from three replicates of each sample.* indicates significant difference between treated and control (*p* < 0.05).

The dry weight (DW) of all the fungal strains grown for 30 days in potato dextrose broth (PDB) and mineral salts broth (MSB) media are shown in Figure 4a,b, respectively. Interestingly, the presence of PEs-rich fraction in both media (treated sample) significantly (*p* < 0.05) enhanced the fungal growth to average values of 2.19 and 2.14 g, respectively, when compared to the fungal growth without PEs-rich fraction (control sample) with values of 0.24 and 0.13 g, respectively. Both *T. harzianum* and *C. cladosporioides* showed higher fungal growth than the other fungal strains. The presence of PEs in the media did not cause any inhibition on the fungal growth, indicating the non-toxicity of PEs on these microbes. As observed, the ability of fungal strains to utilize the compounds present in the PEs-rich fraction was clearly demonstrated in PDB medium when there was significant (*p* < 0.05) enhancement in growth, although this medium is an enrichment culture medium of carbon source [23]. On the other hand, growth was lower in MSB due to the absence of other organic carbon sources.

The ability to grow in the media containing PEs augurs well with the ability to degrade the PEs. However, the enhancement of fungal growth could be the additive effect of both PEs and other nutrients present in the rich fraction. Lipase and esterase activity could be involved in both PE degradation and the oil components present in the fraction. The utilization of the PEs-rich fraction by some fungi has been shown to be due to their ability to produce degradative enzymes [9,19,22]. In addition, some fungal strains have been reported to grow and survive on different compounds and conditions due to their adaptation for efficient extraction of nutrients and their potential for biodegradation processes [24].

Fungi are considered as heterotrophic microorganism that can utilize a carbon source from a diverse range of organic substrates [25]. In the present study, PEs-rich fraction seemed to be a suitable substrate for enhancing the growth of the fungal strains tested when added to the PDB and MSB media. However, PEs from *Jatropha* kernel have been reported to be detrimental against some fungal species (*Fusarium* spp., *Aspergillus niger* and *Curvularia lunata*) and bacteria (*Streptococcus pyogenes*, *Proteus mirabilis* and *Pseudomonas putida*) and hence they are utilized as antimicrobial agents for agricultural applications [26].

### 2.4. Cytotoxicity Assay

Table 2 shows the cytotoxic activity of extracts from the control broth containing PEs-rich fraction (without fungal treatment) and fungal extracts of cultures containing PEs-rich fraction. The extract from the control medium showed less than 1% of cell viability indicating the strong cytotoxicity of PEs on both Chang liver and NIH 3T3 fibroblast cell lines. The cytotoxic effects of PEs were consistent with the results reported earlier which show a similar effect for PEs present in the *Jatropha* meal on Chang and Vero cell lines [2]. However, PEs extracted from *J. curcas* meal were also shown to be cytotoxic to breast (MCF-7) and cervical (HeLa) cancer cell lines in a dose-dependent manner [27]. The inhibition of cell viability is related to PE activity, where they may activate protein kinase C in different pathways that induce apoptosis, inflammation and tumorgenesis with impairment of cellular systems. Types of cell lines and PE derivatives have been reported to affect the different mechanism of PE cytotoxic action [2].

On the other hand, dimethyl sulfoxide (DMSO) extracts from the treated PEs with different fungal strains for 30 days showed high cell viabilities ranging from 84.3% to 96.5% as compared to the PEs control. However, significant differences (*p* < 0.05) in percentage viabilities were observed among fungal strains. Notably, extract from cultures of *T. harzianum* strains showed the highest cell viability values. The significant difference (*p* < 0.05) in the percentage of cell viabilities of different fungal treatments reflects the difference in the percentage reduction of PEs among fungal strains. As shown in Table 1, the reduction in PEs was the highest by *T. harzianum* JQ350879.1, *T. harzianum* JQ517493.1 and *P. sinensis* JQ350881.1 and this fits well with the high cell viabilities by the same fungal strains (Table 2). Hence, the observation clearly indicates that the toxicity levels of PEs on the cell lines depends on the reduction in levels of PEs by the fungal strains. This finding also indicates that transformed products (from PEs degradation) if any, are not toxic to the cell lines tested. Furthermore in this study, PEs-rich fraction from *Jatropha* kernel was prepared at a higher level, more concentrated by 22 times than in previous study [2]. This indicates the ability of the fungal strains to tolerate and reduce higher levels of PEs. *In vitro* cytotoxicity study of treated PEs from *Jatropha* kernel has not been reported.

The fungal strains used in the present study were non toxic to both Chang liver and NIH 3T3 cell lines. As shown in Table 3, DMSO culture extracts with different fungal strains incubated at 28 °C for 7 days, did not exhibit any cytotoxic activity against both cell lines at concentration 250 μg/mL. Cell viability ranged from 98.2% to 102.4% and 97.5% to 103.2% for Chang liver and NIH 3T3 cells, respectively, as compared to controls (102.5%, 103.5%, respectively). There was no significant difference (*p* > 0.05) of cell viabilities among fungal strains compared to untreated cell as control for both cell lines. This result indicates these fungal strains are non toxic to both human (Chang liver) and mouse (NIH 3T3 fibroblast) cell lines. A similar observation was reported that *Trichoderma* sp., *Paecilomyces* sp., *Cladosporium* sp. *and Fusarium* sp. did not have an impairment effect on cell line proliferations [28–31]. It is worth noting that Chang liver cells from humans show similar sensitivity to NIH 3T3 fibroblast cells from mouse to either PEs or fungal extracts, indicating the biological similarity between the two cell lines.

## 3. Experimental Section

### 3.1. *Jatropha curcas* L. seeds

Ripe *J. curcas* L. seeds were freshly collected from the Malaysian Agricultural Research and Development Institute (MARDI), Serdang farm in July 2011. The shells were removed and the kernel was ground using a laboratory food grinder (Guangzhou Xulang Machinery, Guangzhou, China) and air dried at room temperature. The weight of dried ground kernel was recorded.

### 3.2. Preparation of Phorbol Esters-Rich Fraction

The dried ground kernel was extracted with methanol in a sealed container at a ratio of 1:3 (*w*/*v*) at room temperature for 15 min. The sample was then centrifuged for 8 min at 3200× *g* to collect the supernatant. Methanol extraction was repeated three times. The solvent was removed by using a rotary evaporator at 65 °C to recover the oil. The amount of oil obtained was 120 g/kg dry matter of kernel. The oily fraction containing PEs was re-extracted with a mixture of diethyl ether and water (1:1) at a ratio of 1:3 (*w*/*v*) in a separating funnel at room temperature [32]. The upper solvent layer containing PEs was separated from the lower phase containing the oil. The PEs-rich fraction was concentrated using a rotary evaporator to remove all the solvents. The amount of PEs-rich fraction obtained was 18 g/kg dry matter of kernel and was thoroughly mixed before use.

### 3.3. Detection of PEs

Phorbol esters-rich fraction was separated using an analytical column (LiChrospher 100, C18, 250 × 4 mm I.D and 5 μm particle size, Merck) on a Surveyor HPLC binary pump with a surveyor diode array detector (DAD) (200–600 nm range; 5 nm bandwidth). Gradient mobile phase comprising deionized water with 0.5% acetic acid (solvent A) and 100% acetonitrile (solvent B) in the ratio from 40:60 (*v*/*v*) until 100:0 (*v*/*v*) of A:B over 50 min at a flow rate of 1.3 mL/min and at room temperature. After going through the detector, the flow was split to allow only 300 μL/min of eluent into the electrospray ionization (ESI) source of MS (liquid chromatography-diode array detector-electrospray ionization mass spectrometry, LC-DAD-ESIMS). ESI-MS analysis was performed on a ThermoFinnigan model LCQ^DECA^ (San Jose, CA, USA) ion-trap mass spectrometer. The hyphenated system was supported with an Xcalibur 1.2 software. The negative ion mass spectra were obtained from the LCQ^DECA^ ESI/MS detector on full ion scan mode (scanning range 100–2000 amu) at a scan rate of 0.5 Hz with the capillary temperature set at 275 °C [1].

### 3.4. Microorganism and Inoculum Preparation

*Trichoderma harzianum* JQ350879.1, *T. harzianum*. JQ517493.1, *P. sinensis* JQ350881.1, *C. cladosporioides* JQ517491.1, *F. chlamydosporum* JQ350882.1, *F. chlamydosporum* JQ517492.1 and *F. chlamydosporum* JQ350880.1 were used in this study. The strains were provided by the Saudi Fungi Center, Taif University. The *Trichoderma* spp. were isolated from soil, while the endophytic fungi were isolated from *Achillea fragrantissima* plant. All seven fungal strains were selected in terms of non-pathogenic and non-toxic effect on humans and animals [11,33]. The pure fungal strains were preserved in potato dextrose agar (PDA) (Oxoid, Basingstoke, UK) slant and stored at −20 °C [34]. Glycerol stock was prepared for the maint of the fungi on PDA and stored at 4 °C. Fungal strains were transferred on PDA plates, incubated at 28 °C for 7 days before used. One 5-mm agar plug from each strain in the PDA plates was estimated to contain around 1–5 × 10^5^ spores/mL by using the serial dilution counting chamber method.

### 3.5. Degradation of PEs

One liter of potato dextrose broth (PDB) containing 4 g potato extract and 20 g dextrose was prepared according to the instruction manual (Oxoid, Basingstoke, UK). The pH of medium was adjusted to pH 5.5 with 12 M hydrochloric acid or 10 M sodium hydroxide. The PEs-rich fraction was mixed with 30 mL PDB in a ratio 1:15 (*w*/*v*) to obtain a concentration of 101.9 mg PEs in 250 mL glass bottles (Schott, Elmsford, NY, USA) and autoclaved. Two plugs (5-mm) of each fungus grown on PDA plates at 28 °C for 7 days were inoculated into each bottle (treatment). Untreated PEs control (without fungus) and PEs treated (with fungus) were incubated for 7, 14, 21 and 30 days at 28 °C. The samples were freeze dried at −45 °C under vapor pressure of 0.129 mBar (Thermo Electron Corporation Modulyod, Newington, NH, USA). The PEs residues were extracted with 5 mL methanol and filtered using a syringe filter (0.22 μm) for quantitative analyses.

The concentration of PEs in the sample was determined using phorbol-12-myristate 13-acetate (PMA) as the standard by HPLC (Agilent Technologies-1200, Waldbronn, Germany) [15] analysis. A standard PMA calibration curve was prepared in a concentration range of 20–100 μg/mL with five different levels. The HPLC analytical conditions (flow rate and column) were the same as described above. The sample injection volume was 20 μL. The gradient was a combination of deionized water (solvent A) and absolute acetonitrile (solvent B) and increased from 40:60 (*v*/*v*) to 100:0 (*v*/*v*). The peak detection was at the UV wave length of 280 nm and the elution time was approximately 25 min.

### 3.6. Utilization of PEs-Rich Fraction as Carbon Source for Fungal Growth

In a separate experiment, fungal growth in two different media was determined to evaluate the ability of fungal strains to utilize PEs-rich fraction as a carbon source. Potato dextrose broth (PDB) was used as the conventional carbon source and mineral salts broth (MSB) was formulated by replacing the carbon source with minerals. The MSB medium contained 0.005 g of NaCl, 1.0 g of NH_4_NO_3_, 0.002 g of FeSO_4_ 7H_2_O, 0.002 g of ZnSO_4_ 7 H_2_O, 0.7 g of K_2_HPO_4_, 0.7 g of KH_2_PO_4_, 0.7 g of MgSO_4_ 7 H_2_O and 0.001 g of MnSO_4_ (Difco Laboratories, Detroit, MI, USA) in one liter of deionized water [35]. Control media were without the PEs-rich fraction, while treated media contained the PEs-rich fraction in a ratio of 1:15 (*w*/*v*). All media were inoculated with two plugs (5-mm) of the seven fungal strains separately and incubated for 30 days at room temperature under shaking (150 rpm) conditions [36]. The fungal dry weight (DW) produced in PDB and MSB media by each fungal strain was aseptically filtered through a glass microfiber filter (Schleider and Schuell GF50, Dassel, Germany) and washed twice with 10 mL of hot distilled water. The biomass was determined gravimetrically after drying the filters in an oven at 80 °C to a constant weight.

### 3.7. Cytotoxicity Assay

A cytotoxicity assay was carried out using Chang liver (human hepatocytes, CCL-13) and NIH 3T3 (Swiss mouse fibroblasts, CRL-1658) cell lines obtained from the American Type Culture Collection (ATCC). After 30 days incubation, the cytotoxic activity of PEs-rich fraction with fungal strains (treated), as well as untreated PEs-rich fraction (control), was evaluated for each fungal strain. A 30 mL PDB sample in a 250 mL Schott bottle was inoculated with two plugs (5-mm) of each fungal strain and incubated at 28 °C for 7 days to evaluate fungal cytotoxicity. All samples of untreated or treated PEs-rich fraction and fungal cultures were freeze dried under antiseptic condition (−40 °C and vapor pressure of 0.129 mBar). Dimethyl sulfoxide (DMSO) in the ratio 1:100 (*v*/*w*) was added to the dried residues to prepare DMSO extract (stock solution). A syringe filter was used to filter the extract. Each DMSO extract at 250 μg/mL was used for the assay. Cells were seeded into 96-well micro-culture plates (5 × 10^3^/100 μL) in Dulbecco’s Modified Eagle Media (DMEM) after treatment with 0.25% trypsin. The cells were incubated at 37 °C in a humidified atmosphere containing 5% CO_2_ for 24 h. Thiazolyl blue tetrazolium bromide dye (MTT) was used to estimate the cell viability [2].

### 3.8. Statistical Analysis

Analysis of variance (ANOVA) was used to analyze the data and mean values were tested by Dunnett’s Multiple Range test at *p* < 0.05. All statistical analyses were done by Graph Pad Prism 5 software (GraphPad Software Inc., San Diego, CA, USA).

## 4. Conclusions

In conclusion, the present study demonstrated that all the seven fungal strains were able to remove the four phorbol ester derivatives extracted from local *J. curcas* Linn. kernel in the range of 88.9%–99.7% after 30 days of incubation. The increase in fungal growth in PDB and MSB media containing the PEs-rich fraction clearly showed the ability of the fungi to utilize the PEs as well as other nutrients present in the fraction. The cytotoxic activity of the fungal extracts grown in medium containing the PEs-rich fraction was markedly decreased in accordance with the percentage removal of PEs. The strains, notably *T. harzianum* JQ350879.1, *T. harzianum* JQ517493.1 and *P. sinensis* JQ350881.1 could be promising biological agents to remove the toxic PEs present in the *J. curcas* kernel.

## Figures and Tables

**Table 1.** Percentage degradation of phorbol esters (PEs) by fungal strains in potato dextrose broth (PDB) medium at different incubation times.

Fungal strains	Percentage loss of PEs			

	7 days	14 days	21 days	30 days
*T. harzianum* JQ350879.1	28.4 a,v ± 1.44	51.0 b,v ± 0.80	90.0 c,v ± 0.27	99.7 d,v ± 0.01
*T. harzianum* JQ517493.1	23.4 a,v ± 0.25	47.6 b,w ± 0.57	88.8 c,v ± 0.34	99.4 d,v ± 0.05
*P. sinensis* JQ350881.1	24.9 a,v ± 1.96	45.3 b,x ± 0.33	86.2 c,w ± 0.38	98.9 d,v ± 0.02
*C. cladosporioides* JQ517491.1	16.4 a,w ± 0.88	42.9 b,y ± 0.20	78.0 c,x ± 0.15	96.9 d,w ± 0.03
*F. chlamydosporum* JQ350882.1	13.7 a,w ± 1.97	33.6 b,z ± 0.27	70.1 c,y ± 0.50	92.2 d,x ± 0.40
*F. chlamydosporum* JQ517492.1	11.5 a,w ± 1.97	34.8 b,z ± 1.23	69.8 c,y ± 0.24	89.1 d,y ± 0.29
*F. chlamydosporum* JQ350880.1	13.1 a,w ± 1.97	33.6 b,z ± 0.27	66.9 c,z ± 0.21	88.9 d,y ± 0.60

Mean values of three replicates ± standard error.

a,b,c,d, letters show significant difference (*p* < 0.05) within row.

v,w,x,y,z show significant difference (*p* < 0.05) within column.

**Table 2.** Percentage of cell viability of Chang liver and NIH 3T3 cell lines exposed to 250 μg/mL of dimethyl sulfoxide (DMSO) extract of fungal cultures containing PEs-rich fraction after 30 days incubation.

Fungal treated PEs	Cell viability (%)	

	Chang	NIH3T3
Untreated PEs (control) 1	0.4 c,d ± 0.07	0.3 c,d ± 0.06
*T. harzianum* JQ350879.1	96.5 a,d ± 0.57	93.1 a,d ± 0.64
*T. harzianum* JQ517493.1	90.5 a,b,^d^ ± 0.64	90.0 a,b,^d^ ± 1.40
*P. sinensis* JQ350881.1	89.1 b,d± 2.30	90.1 a,b,^d^ ± 3.30
*C. cladosporioides* JQ517491.1	89.8 b,d ± 2.38	92.5 a,b,^d^ ± 0.56
*F. chlamydosporum* JQ350882.1	88.6 b,d ± 2.29	89.2 a,b,^d^ ± 0.71
*F. chlamydosporum* JQ517492.1	84.3 b,d ± 4.42	88.0 a,b,^d^ ± 1.53
*F. chlamydosporum* JQ350880.1	86.9 b,d ± 2.41	87.4 b,d ± 2.01

1 The extract obtained from medium containing PEs-rich fraction without fungal treatment. Mean values ± standard error of three replicates;

a,b,c Means with different superscripts in the same column are significantly different (*p* < 0.05);

d Means within row are not significantly different (*p* > 0.05).

**Table 3.** Percentage of cell viability of Chang liver and NIH 3T3 cell lines exposed to 250 μg/mL of DMSO extract obtained from fungal cultures without PEs-rich fraction after 7 days.

Fungal extracts	Cell viability (%)	

	Chang	NIH3T3
Control 1	102.5 ± 0.52	103.5 ± 1.20
*T. harzianum* JQ350879.1	99.4 ± 1.17	100.2 ± 0.53
*T. harzianum* JQ517493.1	102.4 ± 1.97	102.8 ± 2.18
*P. sinensis* JQ350881.1	100.1 ± 2.13	103.2 ± 1.70
*C. cladosporioides* JQ517491.1	101.8 ± 1.87	100.7 ± 1.82
*F. chlamydosporum* JQ350882.1	98.4 ± 1.23	100.2 ± 1.15
*F. chlamydosporum* JQ517492.1	101.9 ± 1.49	97.5 ± 1.33
*F. chlamydosporum* JQ350880.1	98.2 ± 1.08	101.4 ± 1.46

1 DMEM medium. Mean values ± standard error from three independent experiments. Means within rows or columns did not differ (*p* > 0.05).
